# The impact of implicit health symbols on probiotic products on purchase intention among gastrointestinal disease patients: evidence from multiple experiments

**DOI:** 10.3389/fnut.2026.1751124

**Published:** 2026-03-27

**Authors:** Xingxing Xu, Xia He, Xueying Shao, Xinling Xie

**Affiliations:** 1 Department of TCM Nursing Clinic, Hangzhou TCM Hospital Affiliated to Zhejiang Chinese Medical University, Hangzhou, Zhejiang, China; 2 Xiang'an Hospital of Xiamen University, Xiamen University, Xiamen, Fujian, China; 3 School of Clinical Medical, North Sichuan Medical College, Nanchong, China

**Keywords:** disease threat, gastrointestinal disease patients, health anxiety, implicit health symbols, nutrition marketing, probiotic products, purchase intention

## Abstract

Previous studies have demonstrated that gastrointestinal disease patients exhibit a pronounced preference for probiotic products. However, the extent to which purchase intention is influenced by implicit health symbols on product packaging remains insufficiently investigated. To address this research gap, this study, grounded in the Health Belief Model, systematically examined the impact of implicit health symbols on gastrointestinal disease patients' purchase intention through three experiments (*N* = 955 participants). The results indicated that compared to standard packaging, probiotic packages featuring implicit health symbols significantly enhanced patients' purchase intention (Study 1). Mechanistic analysis revealed that implicit health symbols operate by inducing health anxiety, which subsequently drives purchase intention (Study 2). Furthermore, under conditions of high disease threat, the health anxiety triggered by implicit health symbols was significantly intensified, thereby further strengthening purchase intention (Study 3). This study provides new empirical evidence regarding the role of implicit health symbols in shaping the consumption behavior of a specific patient group and elucidates the underlying psychological mechanisms. These findings contribute to health marketing theory and provide empirical support for the design of probiotic product packaging by food companies, while also offering valuable insights for policy-making.

## 1 Introduction

Gastrointestinal diseases encompass a broad spectrum of conditions affecting the digestive tract (1, 2), including irritable bowel syndrome (IBS), inflammatory bowel disease (IBD), celiac disease, and gastroesophageal reflux disease (GERD), which range from acute to chronic and significantly impair patients' quality of life (3–8). The global burden is substantial: an estimated 73.2 billion new cases and 2.86 billion affected individuals

were recorded in 2019 (9), with over 40% of the global population suffering from functional gastrointestinal disorders (10). This high prevalence may be attributed to factors such as the increasing consumption of processed foods, unhealthy lifestyles, and psychological stress (11–13). If inadequately managed, certain GI disorders may progress to severe complications such as malnutrition, intestinal obstruction, or colorectal cancer. While standard treatment protocols for gastrointestinal diseases typically, significant inter-individual variability in treatment efficacy exists, leaving many patients with persistent symptoms (14, 15). Consequently, patients frequently seek complementary therapies, with probiotics gaining attention due to their potential to regulate gut microbiota and alleviate gastrointestinal discomfort (16–18).

Probiotics are believed to modulate gut microbiota, enhance host immune function, improve digestive processes, and provide adjunctive support for various chronic conditions (19–21). In inflammatory bowel disease, probiotics may maintain remission by reducing inflammation and preventing antibiotic-associated diarrhea (22, 23). However, the probiotic market is characterized by a vast array of products with differences in microbial strains, doses, and formulations (9, 24, 25), complicating consumer choice and underscoring the need to understand the factors influencing product selection, especially among populations with specific health needs such as gastrointestinal disease patients. In the product selection process, packaging emerges as a critical determinant of consumer behavior. Through textual and visual elements such as color, shape, and symbols, packaging communicates product characteristics, health benefits, and brand value (9, 25, 26). Health-related products often employ implicit packaging cues, such as low-purine labels, plant-based certifications, and sugar-free claims, to attract health-conscious consumers (23, 27). A growing body of recent research has examined how branding and labeling strategies—including visual design elements, nutritional information formats, and product positioning cues—shape consumer food choices in contemporary nutritional contexts (28). This line of inquiry has demonstrated that packaging design operates not merely as a vehicle for factual information but as an active framing device that influences consumers' inferences about product healthiness, quality, and suitability for specific dietary needs (29). In the context of probiotic products, packaging frequently incorporates explicit or implicit health claims and symbols to enhance product credibility and appeal (30). These elements are particularly pertinent for gastrointestinal disease patients, who are more likely to keenly interpret and act upon such cues when selecting products that align with their specific health needs. While the general influence of packaging on consumer behavior is well-documented, the specific impact of non-propositional, design-based health cues—as distinct from regulated explicit claims—on the purchase decisions of health-sensitive populations remains underexplored, particularly in the context of probiotic products. The present study addresses this gap by extending recent work on branding and labeling effects into the domain of implicit health-symbol framing and by examining the psychological mechanisms through which such framing operates among a vulnerable patient population.

In this study, “implicit health symbols” are conceptualized more precisely as implicit health-symbol framing—a coordinated set of packaging cues (iconography, color, typography, layout, and technical identifiers) that suggest medical/health relevance and potential efficacy without making explicit, falsifiable health claims. Consistent with this definition, our experimental manipulation adopts a medical-scientific aesthetic framing as a representative form of implicit health-symbol framing (31, 32). These symbols, commonly used in probiotic marketing, aim to subtly foster positive product associations and strengthen consumer perception of product efficacy (33, 34). Existing research suggests that such implicit symbols may leverage cognitive heuristics, prompting consumers to make broader inferences about product quality based on limited cues (35). This effect may be magnified among gastrointestinal disease patients, whose health-related vulnerability heightens their sensitivity to and valuation of health-related cues. These patients may interpret implicit health symbols as specialized recognitions of symptom relief, thereby increasing their likelihood of purchase. Despite the widespread use of such symbols, the specific mechanisms through which they influence the purchase intentions of gastrointestinal disease patients remain underexplored. This constitutes a significant gap in understanding how implicit health symbols shape product preferences among health-vulnerable populations.

Purchase intention represents the propensity of consumers to acquire a specific product or service and serves as an effective predictor of actual purchase behavior (36–38). For gastrointestinal disease patients, the motivation to purchase probiotics often stems from a strong need for symptom management and a desire for health improvement, differing fundamentally from the motivations of general consumers. Implicit health symbols on packaging may enhance purchase intention by subtly conveying health commitments, increasing product appeal, and simplifying the decision-making process for these patients. These symbols act as heuristic cues, guiding patients toward perceived health-promoting solutions, particularly under conditions of information overload or limited cognitive resources. While prior research has established the role of packaging in general consumer behavior, its specific impact through implicit symbols on the purchase intentions of gastrointestinal disease patients remains understudied.

Grounded in the Health Belief Model, we designed and implemented three rigorous experiments. Our findings demonstrate that, compared to standard packaging, implicit health symbols significantly enhance gastrointestinal disease patients' purchase intention. This effect is mediated by the health anxiety induced by these symbols. Furthermore, under conditions of high perceived disease threat, both the anxiety triggered by the symbols and the subsequent purchase intention are significantly amplified. These contributions extend health marketing and consumer behavior theories by elucidating the psychological mechanisms through which implicit symbols influence a vulnerable population, and offer evidence-based recommendations for probiotic packaging design, corporate marketing strategy, and health marketing regulation.

To provide convergent evidence and establish both internal validity and process understanding, we conducted three studies that build sequentially. Study 1 (main effect). Study 1 tests the core prediction that implicit health-symbol packaging (vs. conventional packaging) influences consumers' purchase intention. This study establishes the basic effect and provides initial evidence that subtle health-related cues embedded in packaging can shape consumer responses. Study 2 (mechanism). Study 2 examines why the effect occurs by testing health anxiety as the proposed mediator linking packaging cues to purchase intention. This study strengthens the theoretical account by identifying the underlying psychological process. Study 3 (boundary condition and moderated mediation). Study 3 tests when the effect is stronger or weaker by introducing disease threat as a moderator and evaluating a moderated mediation model. This study clarifies the boundary condition and demonstrates that the indirect effect through health anxiety depends on situational threat. Together, these studies triangulate the main effect, the mechanism, and the boundary condition, offering a coherent account of how implicit health-symbol packaging influences consumer decision-making.

Our distinction between implicit health-symbol packaging and explicit health/nutrition claims is consistent with how food-labeling regulations and the broader food-labeling literature typically differentiate between propositional claims and non-claim cues. Regulatory definitions most clearly specify requirements for nutrition (nutrient-content) claims and health claims, which are usually verbal or numeric statements that explicitly describe nutrient levels or a relationship between consumption and health outcomes. By contrast, many packaging elements that consumers may interpret as “healthy”—such as icons, imagery, colors (e.g., green), minimalist design, or nature-related visuals—often do not constitute a formal health/nutrition claim on their own, but may still be scrutinized under broader rules concerning misleading or deceptive labeling and marketing communications. Accordingly, we conceptualize implicit health symbols as suggestive cues that convey a health-related meaning without asserting an explicit, testable health proposition. Critically, we recognize that in real-world packaging—and in our experimental stimuli—these cues do not appear in isolation but co-occur as an integrated design ensemble. We therefore operationalize implicit health symbols as a medical-scientific aesthetic framing: a coherent, multi-dimensional packaging gestalt in which iconographic, chromatic, typographic, technical-lexical, and textual elements jointly evoke associations of medical authority and scientific rigor. This operationalization is grounded in semiotic theory's principle that meaning arises from sign systems rather than from isolated signs (39), in Gestalt psychology's demonstration that packaging is perceived as a holistic configuration (40), and in dual-process models' identification of a shared heuristic-processing mechanism that unifies these diverse design elements at the psychological level (41). This boundary is important for two reasons. First, it allows us to investigate a subtle yet prevalent form of marketplace communication that may influence consumers even in the absence of an explicit claim. Second, it clarifies that our findings speak to the psychological impact of an integrated medical-scientific aesthetic framing composed of multiple design-based health inference cues, complementing—rather than duplicating—prior work focused on regulated health and nutrition claims. Third, it acknowledges that the persuasive impact we observe is attributable to the holistic gestalt rather than to any single design element, a distinction with important implications for both marketing theory and regulatory practice.

## 2 Literature review and theoretical derivation

### 2.1 The cognitive mechanisms of implicit health symbols and conventional packaging design

The implicit health symbols in packaging design play a pivotal role in shaping consumer cognition, grounded in the theoretical foundations of sensory marketing and the Heuristics—Systems model (41, 42). Implicit health symbols typically refer to non-verbal visual or tactile elements that subtly activate consumers' mental schemas related to “naturalness,” “scientific efficacy,” or “medical-grade” quality through metaphorical and metonymic mechanisms (39, 43). Under conditions of limited cognitive resources, particularly in situations of low product involvement, consumers tend to rely heavily on these peripheral cues for quick judgments (44, 45). This heuristic processing pathway, based on implicit health symbols, contrasts distinctly with the central route processing that requires in-depth analysis of textual information.

In comparison, conventional packaging design often employs relatively neutral elements, such as standard typography, basic color blocks, and generic graphics devoid of specific health metaphors. Its information transmission mechanism relies more on central route processing, requiring consumers to actively decode textual information to assess product value (46). According to the Health Belief Model (47), consumers' evaluation of conventionally packaged products is strongly dependent on the perceived benefits of the product in relation to their specific health needs, moderated by perceived barriers (48). While research indicates that conventional packaging can trigger more rational decision-making processes among highly knowledgeable and motivated consumers (49), ordinary consumers, particularly in the domain of specialized health products, often face significant evaluation difficulties due to a lack of professional knowledge (50), potentially leading to delayed decisions or reliance on simplified cues.

Patients with gastrointestinal disorders constitute a unique demographic that may be particularly sensitive to implicit health symbols, driven by psychological mechanisms that extend beyond those of ordinary consumers. Heightened health anxiety (51) and amplified somatic perception (52) place this group in a state of heightened surveillance for disease threats (53), resulting in significant attentional bias toward health-related cues (54). Attribution theory suggests that these patients are more likely to attribute vague somatic discomfort to deteriorating gastrointestinal conditions, thereby intensifying their motivation to seek solutions (55). In this context, implicit health symbols activate cognitive schemas related to health recovery or scientific intervention (56), providing immediate psychological comfort and a sense of control (43), and potentially triggering placebo effects (57). Notably, the concept of gut microbiota is particularly abstract for ordinary consumers (58), making suggestive symbols such as bacterial microscopic images or cross-sectional diagrams of the gut act as cognitive anchors for understanding these complex mechanisms (59). Empirical evidence supports this notion; for instance, Labus et al. (60) found that IBS patients exhibited significantly higher preference for and positive evaluation of implicit health symbols such as “gentle” and “natural” compared to healthy controls, reflecting the strong emotional drivers in their decision-making processes.

The operational definitions distinguishing the experimental group's implicit health symbol packaging from the control group's conventional packaging across five design dimensions are summarized in Table 1, with the corresponding design rationale elaborated in Appendix A **“Material of stimulation”**.

**Table 1:** Operationalization of implicit health-symbol framing (medical-scientific aesthetic) vs. conventional packaging across design dimensions.

Dimension of design	Experimental group: implicit health-symbol framing (medical-scientific aesthetic design)	Control group: conventional wellness/nature aesthetic packaging
Core icon	Bacterial micrograph/schematic of intestinal anatomy	Illustrations of universal probiotics (abstract flora, natural plants)
Color scheme	Cool colors (blue and green) symbolize medicine and technology	Warm colors (orange and yellow) symbolize nature and everyday life
Mark of specialty	Strain number: lactobacillus ABC-1	Contains active probiotics
Typographic language	Laboratory report style (border, grid line)	Simple commercial packaging (no border, large color block)
Text of slogan	Developed with clinical insights for gut support	Take care of digestive health every day

Importantly, these heuristic cues rarely operate in isolation on actual product packaging. Rather, they typically co-occur as an integrated ensemble—a medical-scientific aesthetic framing—whose persuasive impact derives from the synergistic interaction of multiple design dimensions rather than from any single symbolic element.

#### 2.1.1 Conceptual delimitation: from explicit claims to implicit health symbols as medical-scientific aesthetic framing

A critical conceptual issue in the study of health-related packaging concerns the boundaries among distinct categories of packaging communication. To ensure analytical precision and to situate our experimental operationalization within a rigorous theoretical framework, we distinguish three tiers of health-related packaging communication that occupy different positions along a continuum from propositional explicitness to associative implicitness.

At one end of this continuum lie explicit health and nutrition claims—propositional, typically text-based assertions that state a specific relationship between product consumption and a health outcome or that describe a product's nutrient content in quantitative terms. These claims are falsifiable, subject to regulatory scrutiny under frameworks such as the European Union's Regulation (EC) No 1924/2006 or the U.S. FDA's qualified health claim framework, and require scientific substantiation prior to market authorization (61). Their persuasive mechanism operates primarily through the central processing route, as they invite deliberative evaluation of propositional content (45). At the opposite end of the continuum lies conventional or neutral packaging, which employs standard commercial design conventions—generic color palettes, basic typography, universal product imagery—that do not carry specific health metaphors, medical connotations, or scientific associations. Conventional packaging communicates product identity and category membership without activating health-related cognitive schemas. Between these two poles lies the intermediate category that constitutes the focus of the present investigation: implicit health symbols. However, the term “symbols” might suggest isolated, discrete semiotic units, whereas our experimental operationalization integrates multiple co-occurring design dimensions—iconographic, chromatic, typographic, technical-lexical, and textual-framing —into a unified packaging treatment. We argue, drawing on three converging theoretical traditions, that this integration is not an operationalization weakness but rather a theoretically principled reflection of how implicit health symbols function in real-world packaging and in consumer cognition.

First, from the perspective of semiotic theory, meaning in commercial packaging does not reside in individual, isolated signs but emerges from sign systems—structured constellations of co-occurring semiotic elements that jointly constitute a coherent semiotic field. In Barthesian terms, the individual design elements (micrographs, cool tones, laboratory typography) function as first-order signifiers whose second-order connotative meaning—“medical-scientific authority,” “clinical efficacy,” “professional-grade intervention”—arises precisely from their co-presence and mutual reinforcement within the same communicative artifact. The medical-scientific meaning is therefore a property of the system, not of any single element in isolation. This principle of semiotic synergy provides the theoretical warrant for treating the multiple design dimensions as constituents of a single higher-order construct—what we term the medical-scientific aesthetic framing.

Second, from the perspective of Gestalt principles of perceptual organization, consumers do not process individual packaging elements in isolation and then aggregate their impressions additively. Rather, they perceive packaging as a unified perceptual whole in which the emergent holistic impression may qualitatively differ from the sum of its constituent parts. In the context of our stimuli, the medical-scientific impression is an emergent perceptual property that arises from the synergistic configuration of multiple design dimensions. This Gestalt perspective further justifies operationalizing implicit health symbols as an integrated multi-dimensional treatment: decomposing the gestalt into its constituent elements would alter the very perceptual experience under investigation and would sacrifice the ecological validity that characterizes real-world packaging encounters.

Third, from the perspective of the Heuristic-Systematic Model and dual-process theories of persuasion, the multiple design elements in our stimuli share a common psychological mechanism: they all function as peripheral or heuristic cues that bypass deliberative, propositional evaluation and instead trigger rapid, schema-driven associative inferences about product quality and efficacy. Whether a consumer encounters a bacterial micrograph, a cool blue-green color scheme, or a laboratory-style typographic layout, the processing pathway is fundamentally the same—non-deliberative activation of a “medical-scientific competence” schema that enhances perceived product credibility without requiring evaluation of any explicit, falsifiable health proposition. This shared mechanistic basis—heuristic processing via schema activation—constitutes the functional unity that binds these diverse design elements into a single conceptual category and distinguishes them categorically from explicit claims, which engage central-route processing.

The term implicit health symbols serves as the overarching theoretical construct, defined as non-propositional packaging elements that suggest health benefits through associative, schema-activating mechanisms without asserting falsifiable health propositions. In the present study, this construct is operationalized as a medical-scientific aesthetic framing—a coherent, multi-dimensional packaging gestalt composed of iconographic, chromatic, typographic, technical-lexical, and textual-framing elements that jointly evoke associations of medical authority, scientific rigor, and clinical efficacy. This operationalization is distinguished from explicit health claims (which are propositional and engage central-route processing) and from conventional packaging (which lacks health-specific connotative content).

A medical-context recommendation can function as a strong contextual cue that increases baseline perceived legitimacy and reduces uncertainty about category appropriateness. Importantly, such a cue may also amplify the impact of packaging aesthetics: when consumers are already primed to view probiotics as medically relevant (e.g., via physician-context information), medical-scientific packaging cues may become more diagnostic and easier to interpret, thereby strengthening heuristic inferences about efficacy and fit. In this sense, physician-context recommendations may operate as an “authority/legitimacy scaffold” that raises receptivity to medical-scientific design cues, potentially increasing the observed effect sizes relative to purely self-initiated, shelf-based choice contexts.

### 2.2 The impact of implicit health symbols on the purchase intention

Implicit health symbols on probiotic packaging subtly imply potential health benefits through implicit visual or textual elements. From a semiotics perspective (39), these symbols activate health-related cognitive frameworks through metaphor and metonymy, reinforcing associations with naturalness and efficacy that enhance the perceived value of probiotic products (33). More broadly, recent research on food branding and labeling has demonstrated that visual and informational packaging elements exert substantial influence on consumers' health-related inferences, often independent of the factual nutritional content of the product (62). These findings underscore that consumer perceptions of healthiness are shaped not only by what is explicitly stated on a label but also by the overall visual and semiotic impression that packaging conveys. The present study extends this line of inquiry by focusing specifically on a coordinated set of implicit health-symbol cues—operationalized as a medical-scientific aesthetic framing—and by examining their effects among a population whose health vulnerability may render them particularly susceptible to such design-based inferences. For gastrointestinal disease patients, the impact of implicit health symbols may be significantly amplified. This demographic exhibits heightened health sensitivity, leading to attentional bias toward digestive health-related cues (54), and their purchasing behavior is often driven by chronic symptom management needs (63).

The heuristic appeal of implicit health symbols is particularly relevant in this context. Research on functional foods demonstrates that implicit health cues enhance preferences among high-risk consumers, and that packaging with scientific or technological appeal improves perceived therapeutic efficacy by implicitly signaling research support. Attribution theory further suggests that these patients are more likely to attribute positive health expectations to products bearing implicit health symbols, thereby strengthening purchase motivation. As heuristic cues, implicit health symbols can facilitate rapid decision-making through peripheral processing, directly influencing behavioral intentions without requiring extensive deliberative evaluation.

Based on the aforementioned theoretical analysis and empirical evidence, this study proposes the following core hypotheses:

**H1:** compared to conventional packaging, probiotic products featuring implicit health symbols will have a significantly positive impact on the purchase intentions of gastrointestinal disease patients.

### 2.3 The mediating role of health anxiety

Health anxiety refers to individuals' persistent and excessive concern about their health, often manifesting as catastrophic interpretations of somatic symptoms and heightened vigilance toward potential health threats (51). In the gastrointestinal disease patients, this anxiety is particularly pronounced. Chronic gastrointestinal disorders, functional or organic, often involve recurrent, unpredictable symptoms that not only significantly impair quality of life but also perpetuate fear of health uncertainty and concern about disease progression. The Health Belief Model conceptualizes health anxiety as a critical amplifier of perceived disease susceptibility and severity (64, 65), driving individuals to actively seek preventive or alleviating measures (66, 67). In consumer behavior, health anxiety has been identified as a key psychological mechanism that translates external marketing cues into specific action motives (68, 69), with high-anxiety individuals more inclined to choose products whose packaging suggests therapeutic effects (36, 70).

We propose that implicit health symbols activate and transiently elevate health anxiety among gastrointestinal disease patients by serving as reminders of potential gut problems or microbial imbalance (71). This anxiety elevation, rather than being purely maladaptive, may represent an adaptive response that mobilizes individuals to seek solutions (72), with probiotic products positioned by implicit health symbols as accessible tools for relief. Health anxiety thus mediates the relationship between implicit health symbols and purchase intention: symbols first elevate anxiety, which in turn drives purchasing as a compensatory strategy to address perceived threats. Within the HBM framework, implicit health symbols serve as external cues to action that amplify the perceived benefits of the product among anxious individuals, ultimately leading to stronger purchase intentions.

Based on the aforementioned theoretical derivation and empirical evidence, this study proposes the following hypothesis:

**H2:** health anxiety mediates the impact of implicit health symbols on the purchase intentions of gastrointestinal disease patients.

### 2.4 The moderating role of perceived disease threat

Perceived disease threat refers to individuals' subjective assessment of the health risks associated with their condition, typically encompassing perceptions of disease severity, controllability (73, 74), likelihood of recurrence, and potential consequences. For gastrointestinal disease patients, high perceived disease threat primarily stems from the unpredictability of chronic symptoms and/or the risk of serious complications (75, 76). Research indicates that high perceived disease threat amplifies individuals' dependency on and reactivity to external cues (77). In the context of probiotic marketing, perceived disease threat may moderate the impact of implicit health symbols on patients' purchase intentions: under high threat conditions, implicit health symbols are more likely to be interpreted as urgent and targeted solutions; whereas under low threat conditions, they may be viewed as merely auxiliary options for daily wellness (78). According to cognitive appraisal theory (79, 80), high perceived disease threat triggers strong primary and secondary appraisals. Implicit health symbols, by conveying professionalism, scientific rigor, and efficacy, enhance patients' perceptions of having “effective coping resources,” thereby interacting with high threat perception to jointly elevate purchase intentions.

The interaction between perceived disease threat and implicit health symbols in influencing purchase intentions operates through the mechanism of threat perception amplifying the signal value and motivational relevance of implicit health symbols. Under conditions of high perceived disease threat, implicit health symbols are more likely to be interpreted by patients as direct, effective interventions for their pressing health issues, thereby significantly enhancing their purchase motivation; whereas, under low threat conditions, this effect is substantially diminished (81). In the context of gastrointestinal diseases, the intensity of this interaction is often driven by the severity of patients' current symptoms. High perceived disease threat may also enhance the potential of implicit health symbols to induce placebo effects, as patients' expectations of improvement are more intense in highly anxious states (82). According to the attentional bias model, high perceived disease threat biases patients' attentional resources, prioritizing the processing of health-related cues in the environment (83). Consequently, under high-threat conditions, implicit health symbols are more likely to capture patients' attention and undergo deeper cognitive processing, thereby more effectively enhancing purchase intentions. In contrast, under low perceived disease threat, patients' decision-making processes may lean more toward rationality, relying on textual information and central route processing, with the influence of implicit health symbols as peripheral cues being relatively limited.

The interaction between perceived disease threat and implicit health symbols also influences levels of health anxiety, reflecting the moderating role of threat perception in the pathway from implicit health symbols to health anxiety. Under high perceived disease threat, exposure to implicit health symbols is more likely to trigger intense anxiety responses in patients. In such cases, implicit health symbols may be catastrophically interpreted as evidence validating patients' worst fears, leading to abrupt spikes in anxiety levels. Conversely, under low perceived disease threat, the same implicit health symbols may elicit only mild or transient anxiety fluctuations, with overall anxiety levels remaining relatively stable. Among gastrointestinal disease patients, this interaction is particularly pronounced due to the frequent overlap between threat perception and current somatic experiences. Implicit health symbols serve as external reminders that, in high-threat contexts, are more likely to exacerbate patients' concerns about symptom meanings and future health outcomes (84). According to cognitive bias theory, high perceived disease threat strengthens individuals' tendencies to interpret ambiguous or neutral information as threatening. As ambiguous health cues, implicit health symbols are more likely to serve as cognitive anchors in high-threat conditions, being assigned negative, catastrophic meanings that amplify anxiety bias. In contrast, under low perceived disease threat, implicit health symbols are more likely to be neutralized or activate only mild positive expectations.

Based on the aforementioned theoretical analysis, this study proposes the following hypotheses:

**H3a:** perceived disease threat positively moderates the impact of implicit health symbols on the purchase intentions of gastrointestinal disease patients.
**H3b:** perceived disease threat positively moderates the impact of implicit health symbols on health anxiety among gastrointestinal disease patients.

To test the boundary condition proposed in H3a–H3b, we required a context that reliably elevates perceived disease threat, i.e., subjective appraisals of severity and susceptibility. In real-world food and supplement markets, perceived disease threat often fluctuates not only with one's chronic symptom trajectory (e.g., flare-ups and recurrence concerns) but also with salient public-health information (e.g., local outbreaks and contamination alerts). Accordingly, Study 3 uses a disease-threat prime to vary perceived severity/susceptibility and examine whether the persuasive impact of medical-scientific packaging cues is amplified when individuals feel more threatened by disease.

### 2.5 Mechanism contingency under varying disease threat

Although we hypothesize that perceived disease threat strengthens the effects of implicit health-symbol framing on both purchase intention (H3a) and health anxiety (H3b), we further theorize that heightened threat can qualitatively alter how the effect unfolds. Drawing on the Health Belief Model, disease threat elevates perceived severity and susceptibility, thereby increasing the motivational relevance of external cues to action. In high-threat contexts, packaging cues that convey medical-scientific competence may be processed less as ambiguous “health-related signals” that primarily elicit worry, and more as actionable coping affordances—that is, readily available means to regain control and reduce perceived risk. Consequently, the persuasive impact of implicit health-symbol framing may become more proximal and action-oriented, yielding a comparatively stronger direct effect on purchase intention.

This anticipated shift is consistent with fear-appeal and coping appraisal accounts (e.g., Protection Motivation Theory and the Extended Parallel Process Model). When perceived threat is high, individuals' responses depend not only on fear arousal but also on coping appraisal (response efficacy and self-efficacy). Medical-scientific aesthetic cues (e.g., clinical color schemes, laboratory-report-like layouts, strain-like identifiers) can implicitly signal higher response efficacy (“this product looks clinically grounded”) and thus facilitate a “danger-control” orientation in which individuals adopt recommended actions more directly, rather than relying on affective reassurance-seeking driven by health anxiety. By contrast, under low threat, the same cues are less urgent and more likely to operate through internal affective states—especially transient health anxiety—because consumers have greater latitude for interpretation, rumination, and emotion-based inference. Therefore, we expect disease threat not only to moderate the strength of effects (H3a–H3b) but also to influence the relative prominence of the indirect (via health anxiety) vs. direct pathway, such that mediation through health anxiety is more pronounced under low threat, whereas the process becomes more direct under high threat.

**Figure 1:**
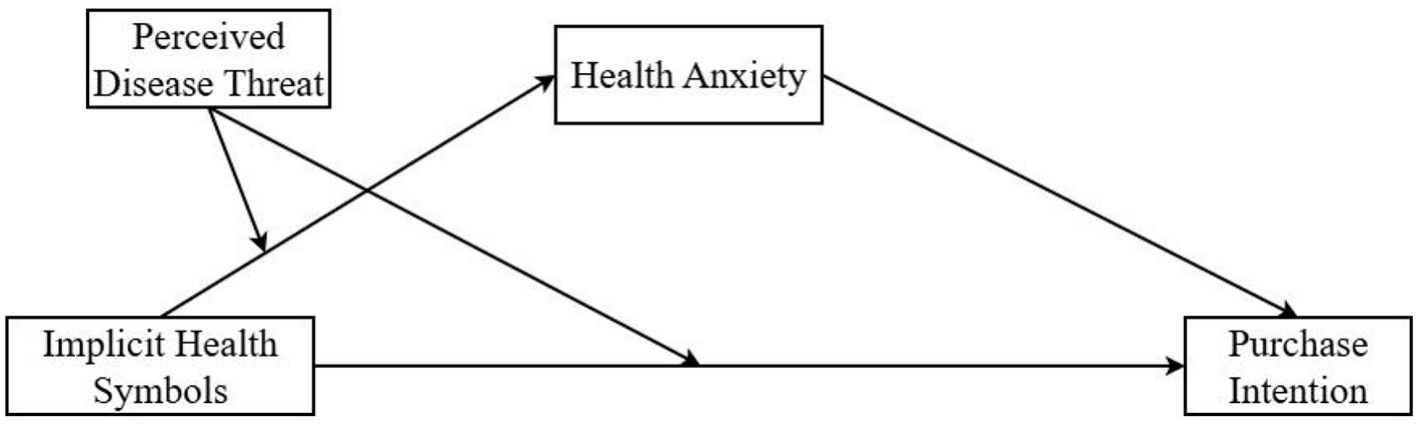
Diagram of the theoretical framework model.

The theoretical framework model of this study, as shown in Figure 1.

## 3 Research overview

To test the four hypotheses proposed above, this study conducted three interrelated online experiments. Specifically, in Study 1, we employed one-way analysis of variance (ANOVA) and confirmed that implied health symbols on probiotic products (vs. conventional packaging) exerted a significant positive effect on purchase intention among patients with gastrointestinal disorders, thereby supporting Hypothesis 1 (H1). In Study 2, using PROCESS Model 4, we demonstrated that health anxiety significantly mediated the effect of implied health symbols (vs. conventional packaging) on purchase intention among patients with gastrointestinal disorders, providing support for Hypothesis 2 (H2). In Study 3, we applied PROCESS Model 8 to examine the interactive effect of disease threat and implied health symbols (vs. conventional packaging) on both purchase intention and health anxiety among patients with gastrointestinal disorders, which supported Hypotheses 3a and 3b.

**Figure 2:**
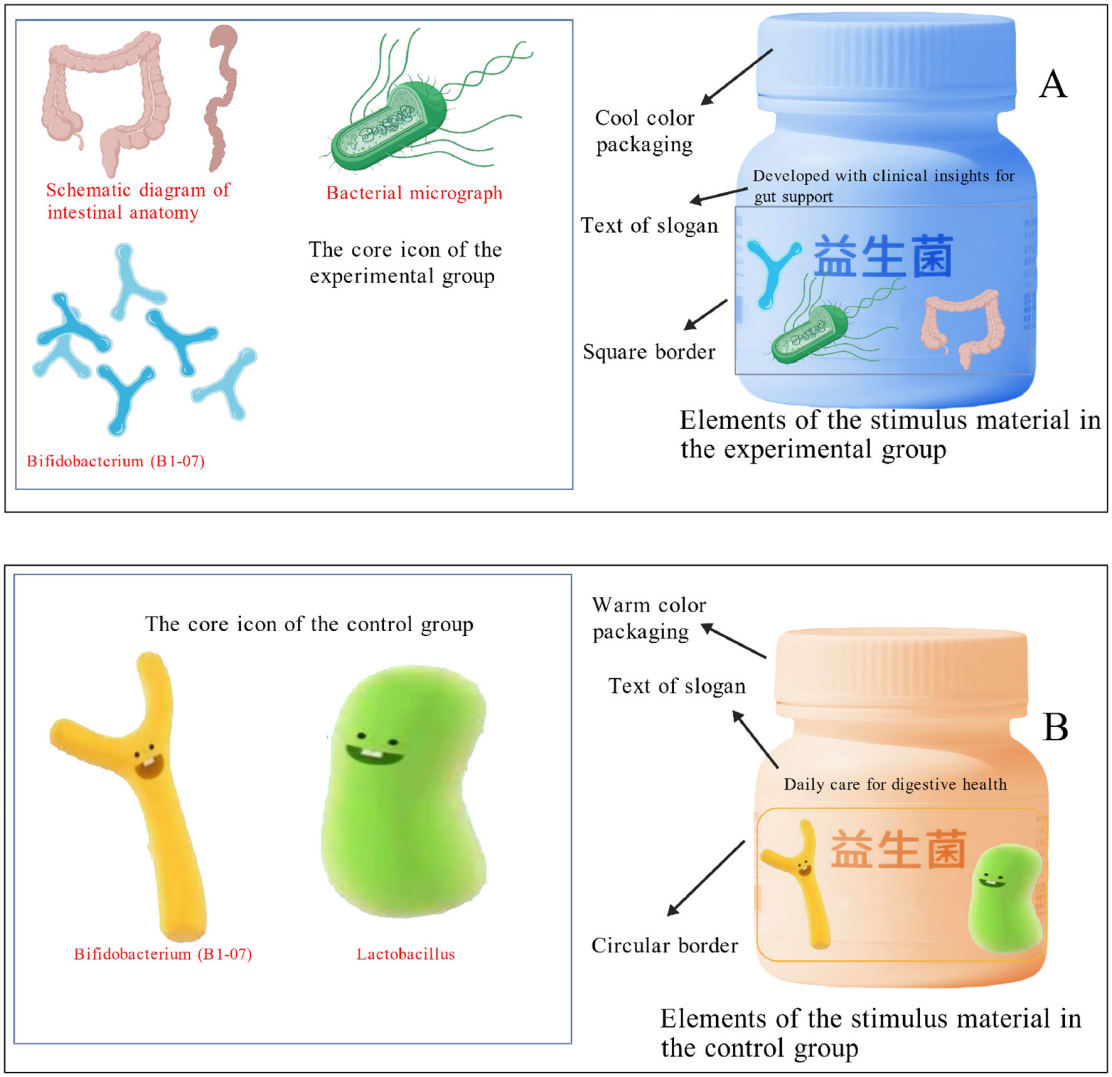
Experimental stimulus maps used in the three studies. **(A)** Elements of the stimulus material in the experimental group. **(B)** Elements of the stimulus material in the control group.

To further enhance the robustness and validity of the experimental manipulations, distinct stimulus descriptions were employed across the three studies. The stimulus images used in all three studies are presented in Figure 2. These images were professionally created using the BioGDP medical illustration platform (https://BioGDP.com) (85). Additionally, established manipulation-check items were administered in each study to verify the effectiveness of the experimental manipulations. Demographic characteristics of the participants across the three studies are summarized in Table 2. The effectiveness of the stimulus material was demonstrated in A pre-experiment, as described in “Experiment S1: A prospective operationalization of implicit health symbols” in Appendix A. The manipulation was designed to operationalize an implicit health-symbol framing in the form of a medical-scientific aesthetic design rather than a single isolated symbol. Specifically, in the medical-scientific framing condition, the package incorporated micrograph-/anatomy-resembling imagery, cool clinical colors, laboratory-report-like layout elements (e.g., grids/borders), and a strain-like alphanumeric identifier. In the conventional condition, the package adopted a more common “everyday wellness/nature” aesthetic (e.g., warm colors and generic natural imagery) without medical-scientific styling. Importantly, neither condition contained explicit therapeutic or disease-risk-reduction claims; thus, the manipulation targets design-based health inference cues (implicit framing) rather than regulated, propositional health claims.

**Table 2:** Demographic information for four experiments.

Variables	Items	Experiment 1		Experiment 2		Experiment 3	
		***N***	**Proportion**	***N***	**Proportion**	***N***	**Proportion**
Gender	Male	116	48.9%	189	52.2%	171	48.0%
	Female	121	51.1%	173	47.8%	185	52.0%
Age	18–25 years old	105	44.3%	83	22.9%	91	25.6%
	26–40 years old	96	40.5%	102	28.2%	94	26.4%
	41–60 years old	19	8.0%	94	26.0%	93	26.1%
	61 years old and above	17	7.2%	83	22.9%	78	21.9%
Educational background	Primary school and below	36	15.2%	76	21%	104	29.2%
	Middle school to high school	101	42.6%	77	21.3%	93	26.1%
	Bachelor degree or above	100	42.2%	149	41.2%	69	19.4%
Monthly income level	≤ 3,000¥	73	30.8%	117	32.3%	84	23.6%
	3,001–6,000¥	94	39.7%	120	33.1%	96	26.9%
	6,001–9,000¥	54	22.8%	94	26.0%	117	32.9%
	9,001¥ and above	16	6.8%	31	8.6%	59	16.6%

Realism and stylisation of the packaging stimuli. To maximize internal validity and isolate the focal construct—implicit health-symbol framing—the packaging stimuli were intentionally designed to create a clear contrast between a medical–scientific aesthetic and a conventional FMCG-like wellness/nature aesthetic. This stylisation is common in experimental packaging research because it increases the signal-to-noise ratio of the manipulation and reduces ambiguity about which semiotic ensemble is being tested. Importantly, our manipulation does not aim to claim that real-world probiotic packages are dichotomously “medical” vs. “ordinary”; rather, it operationalises a theoretically motivated design gestalt that is frequently observed in varying degrees in the marketplace. Nevertheless, we recognize that the degree of visual differentiation between conditions in a controlled experiment may exceed the typical contrast encountered on shelves, which has implications for ecological validity. Thus, our manipulation should be interpreted as a high-contrast instantiation of implicit health-symbol framing intended for causal identification, rather than as a one-to-one replication of any single commercial package currently on the market.

Participants were randomly assigned to conditions, and the only manipulated element was the visual packaging design presented afterward. Thus, any between-condition differences cannot be attributed to differences in the recommendation content itself, but to the packaging-based inference cues.

External relevance to marketplace design practices. Although our stimuli were purpose-built for experimental control, the constituent elements of the medical–scientific framing are consistent with widely documented visual strategies in health and functional-food marketing, where “science/medical” cues are used to signal expertise, efficacy, and credibility (e.g., semiotics-based packaging research and cue-utilization accounts). Thus, the manipulation is externally relevant as an experimentally purified representation of a set of cues that appear—often in milder combinations—across probiotic and broader functional nutrition packaging.

Data were collected via Credamo (www.credamo.com), a professional online research platform widely used in China (86). This platform was selected primarily because patients with gastrointestinal disorders represent a highly specific population with substantial privacy concerns. Credamo's targeted recruitment channels enable precise and efficient access to this hard-to-reach group (87). Moreover, the platform's fully anonymized environment encourages participants to disclose sensitive information regarding their actual medical conditions, associated distress, and genuine purchase intentions, thereby minimizing social desirability bias and discomfort that might arise in face-to-face settings. The reliability and validity of data collected through Credamo have been well-documented in numerous prior marketing and consumer behavior studies (88, 89).

## 4 Study 1: implied health symbols and purchase intention

### 4.1 Method

Study 1 employed a single-factor between-subjects design (probiotic product packaging: implied health symbols vs. conventional packaging) to test the effect of implied health symbols (vs. conventional packaging) on purchase intention among patients with gastrointestinal disorders. The questionnaire was created electronically on Credamo (a professional online data collection platform widely used in China) and distributed to a targeted pool of participants. A total of 250 patients with gastrointestinal disorders were recruited, of whom 13 were excluded due to incomplete responses, yielding a final sample of 237 participants. Each participant received a small monetary compensation (RMB 1). Participants were randomly assigned to either the implied health symbols condition (*n* = 118) or the conventional packaging condition (*n* = 119). Demographic information is presented in Table 1.

Participants in both conditions first read identical scenario descriptions (see Appendix A). The scenario did not recommend any specific brand or packaging style; it only provided a general health-management context. The physician-context information was therefore independent from the subsequent packaging manipulation. Immediately afterward, they were exposed to different product images: participants in the implied health symbols condition viewed Figure 2A, whereas those in the conventional packaging condition viewed Figure 2B. Next, participants completed manipulation-check items measuring perceived health benefits (e.g., “The packaging of the probiotic product shown above makes me believe it can effectively helps resolve gastrointestinal problems”; 1 = strongly disagree, 7 = strongly agree). Purchase intention was then assessed with established items [e.g., “After learning about the probiotic product above, I am willing to purchase it”; 1 = strongly disagree, 7 = strongly agree; adapted from Yang, Gui (101)]. Finally, participants reported their current mood (90) and provided demographic information.

### 4.2 Results

**Manipulation Check**. An independent-samples *t*-test on perceived health benefits revealed that participants exposed to implied health symbols reported significantly higher perceived health benefits (M = 4.53, SD = 1.89) than those exposed to conventional packaging (M = 4.09, SD = 1.47; *t* = 2.01, *p* = 0.046), confirming successful manipulation.

**Main Effect**. A one-way ANOVA with packaging type as the independent variable and purchase intention as the dependent variable showed that purchase intention was significantly higher in the implied health symbols condition (M = 5.14, SD = 1.95) than in the conventional packaging condition (M = 4.28, SD = 1.37; *F*(1, 235) = 15.362, *p* < 0.001, η^2^ = 0.061), supporting Hypothesis 1.

**Covariate Analysis**. Given prior evidence that positive mood can increase purchase intention (91), we conducted an ANCOVA with mood as a covariate. Mood did not significantly affect purchase intention (*F* = 1.70, *p* = 0.194), and the main effect of packaging type remained significant, providing further support for Hypothesis 1.

### 4.3 Discussion

Study 1 demonstrated that patients with gastrointestinal disorders exhibited stronger purchase intention toward probiotic products featuring implied health symbols than toward those with conventional packaging. These symbols appear to serve as extrinsic cues that heighten perceived disease susceptibility and severity, thereby reinforcing the salience of health maintenance. The robustness of this effect was strengthened by ruling out mood as a confounding factor. We propose that the elevated purchase intention may stem from heightened health anxiety in this vulnerable population. Accordingly, Study 2 introduces health anxiety as a mediator.

## 5 Study 2: the mediating role of health anxiety

### 5.1 Method

Study 2 adopted a single-factor between-subjects design (probiotic product packaging: implied health symbols vs. conventional packaging) to examine the mediating role of health anxiety. Using the same Credamo platform, we recruited 370 individuals with gastrointestinal disorders. Each participant received RMB 1 as compensation. After excluding 6 participants for incomplete responses and 2 for failing attention checks, the final sample comprised 362 participants, randomly assigned to the two conditions [Implied health symbols condition (*n* = 180) or the conventional packaging (*n* = 182)]. Demographic information is presented in Table 1.

The procedure mirrored Study 1: participants read the same scenario (Appendix A) before viewing either Figure 2A (implied health symbols) or Figure 2B (conventional packaging). Manipulation checks assessed perceived health/medical emphasis of the packaging (e.g., “The packaging design of the probiotic product you just saw emphasizes its health or medical attributes”; 1 = strongly disagree, 7 = strongly agree). Health anxiety was measured using the 18-item Short Health Anxiety Inventory (Cronbach's α =0.93; sample item: “I am constantly worried about my health”) (92). Purchase intention was assessed with the same scale as in Study 1, followed by demographic questions.

### 5.2 Results

Manipulation Check. Participants in the implied health symbols condition perceived significantly stronger health/medical emphasis (M = 5.42, SD = 1.62) than those in the conventional packaging condition (M = 4.76, SD = 1.21; t = 4.38, *p* < 0.001, Cohen's *d* = 0.46), confirming successful manipulation.

Main Effect. Replicating Study 1, a one-way ANOVA revealed higher purchase intention in the implied health symbols condition (M = 4.82, SD = 1.64) than in the conventional packaging condition (M = 3.96, SD = 1.77; *F*(1, 360) = 23.24, *p* < 0.001, η^2^ = 0.061), again supporting Hypothesis 1.

**Figure 3:**
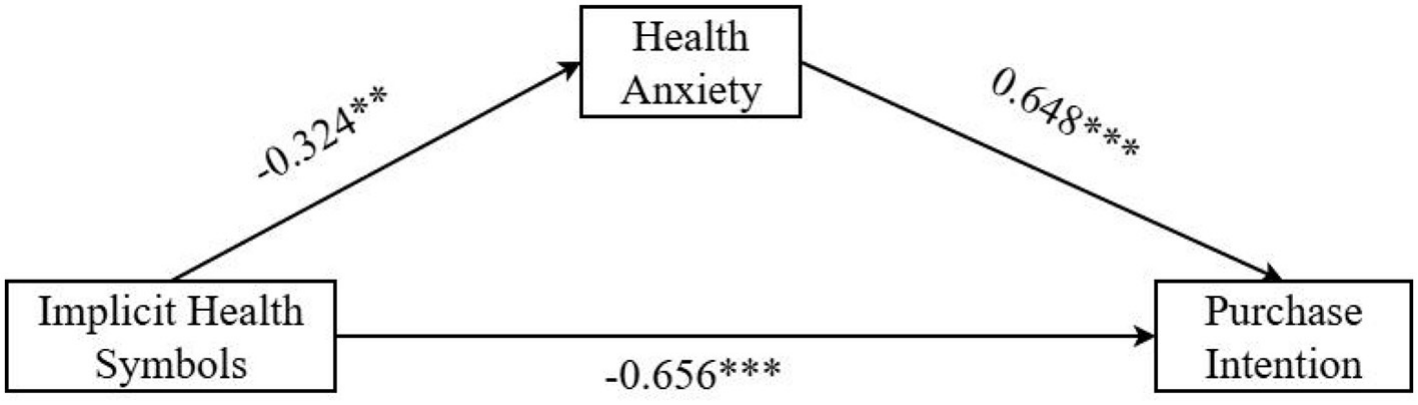
Path coefficient of mediating effect of health anxiety. **p < 0.01; ***p < 0.001.

Mediation Analysis. We tested mediation using PROCESS Model 4 (108) with 5,000 bootstrap samples. Implied health symbols (coded as 1 = implied health symbols, 0 = conventional packaging) significantly predicted health anxiety (β = −0.324, SE = 0.11, 95% CI [−0.548, −0.101], *p* = 0.005) and directly predicted purchase intention (β = −0.656, SE = 0.17, 95% CI [−0.983, −0.329], *p* < 0.001). Health anxiety significantly predicted purchase intention (β = 0.648, SE = 0.08, 95% CI [0.498, 0.797], *p* < 0.001). The indirect effect through health anxiety was significant (β = −0.210, SE = 0.08, 95% CI [−0.369, −0.066]), supporting Hypothesis 2 (see Figure 3).

Covariate Analysis. Gender had no significant effect on purchase intention (*F*(1, 360) = 0.92, *p* =0.337), further confirming the robustness of the findings.

### 5.3 Discussion

Study 2 confirmed that health anxiety mediates the relationship between implied health symbols on probiotic packaging and purchase intention among patients with gastrointestinal disorders. Implied health symbols appear to act as conditioned stimuli that trigger health anxiety by associating with prior aversive experiences, prompting compensatory consumption as a ritualized safety-seeking behavior. Although these findings elucidate the underlying mechanism, they do not address potential boundary conditions. Therefore, Study 3 introduces perceived disease threat as a moderator of the mediated path.

## 6 Study 3: the moderating role of disease threat

### 6.1 Method

Study 3 adopted a 2 (probiotic product packaging: implied health symbols vs. conventional packaging) × 2 (disease threat: high vs. low) between-subjects factorial design to examine the moderating effect of disease threat. Participants were recruited through the professional data collection platform Credamo, with 360 patients with gastrointestinal disorders taking part in the experiment; each received RMB 1 as compensation. Four participants were excluded because they failed the attention check, leaving a final sample of 356. All participants were randomly assigned to either the implied health symbols condition (*n* = 178) or the conventional packaging condition (*n* = 178).

Participants first read the same scenario description used in the previous studies (see Appendix A). Immediately afterward, those in the implied health symbols condition were shown the probiotic product package featuring intestinal icons, whereas those in the conventional packaging condition were shown the package with textual descriptions of intestinal intervention (see Figure 2).

Disease-threat manipulation. We manipulated perceived disease threat by exposing participants to a short public-health news vignette designed to shift subjective perceptions of severity and susceptibility regarding gastrointestinal infectious risk. Importantly, the vignette was written to emphasize health risk and disease uncertainty (e.g., likelihood of infection, symptom burden, and limited controllability) rather than existential themes or death-related content. Participants in the high-threat condition read an outbreak-related vignette highlighting elevated infection risk and severe gastrointestinal symptoms, whereas participants in the low-threat condition read a vignette describing a relatively contained environmental health issue with lower perceived personal risk (see Appendix A). This approach is intended to manipulate perceived disease threat rather than mortality salience. Participants then completed manipulation-check items for packaging conventionality (e.g., “The packaging of the probiotic product shown above appears markedly different from ordinary probiotic products,” 1 = strongly disagree, 7 = strongly agree). Finally, they completed the measures of health anxiety, purchase intention, and demographic information.

Variable coding and interpretation. In Study 3, packaging type was dummy-coded as *X* = 0 for the implicit health-symbol packaging and *X* = 1 for the conventional packaging. Disease threat was dummy-coded as *W* = 0 for the high-threat condition and *W* = 1 for the low-threat condition. Purchase intention and health anxiety were measured on 7-point scales, with higher scores indicating stronger purchase intention and greater health anxiety, respectively. Therefore, a positive regression coefficient indicates that the predictor is associated with an increase in the outcome, whereas a negative coefficient indicates an decrease in the outcome relative to the reference category (i.e., *X* = 0 or *W* = 0). To facilitate non-technical interpretation, we report conditional effects for each experimental cell and visualize the interaction patterns.

### 6.2 Results

For the manipulation check, perceived conventionality served as the dependent variable in an independent-samples t-test. Participants exposed to implied health symbols reported significantly lower perceived conventionality (*M* = 6.00, SD = 1.551) than those exposed to conventional packaging (*M* = 5.49, SD = 1.194; *t* = −3.484, *p* < 0.001), confirming that the packaging manipulation was successful.

The main effect analysis replicated previous studies: purchase intention was significantly higher for probiotic products featuring implied health symbols (*M* = 4.47, SD = 1.801) than for those with conventional packaging (*M* = 3.88, SD = 1.790, *F*(1,354)=9.603, *p* = 0.002, η^2^ = 0.026), again supporting Hypothesis 1.

**Figure 4:**
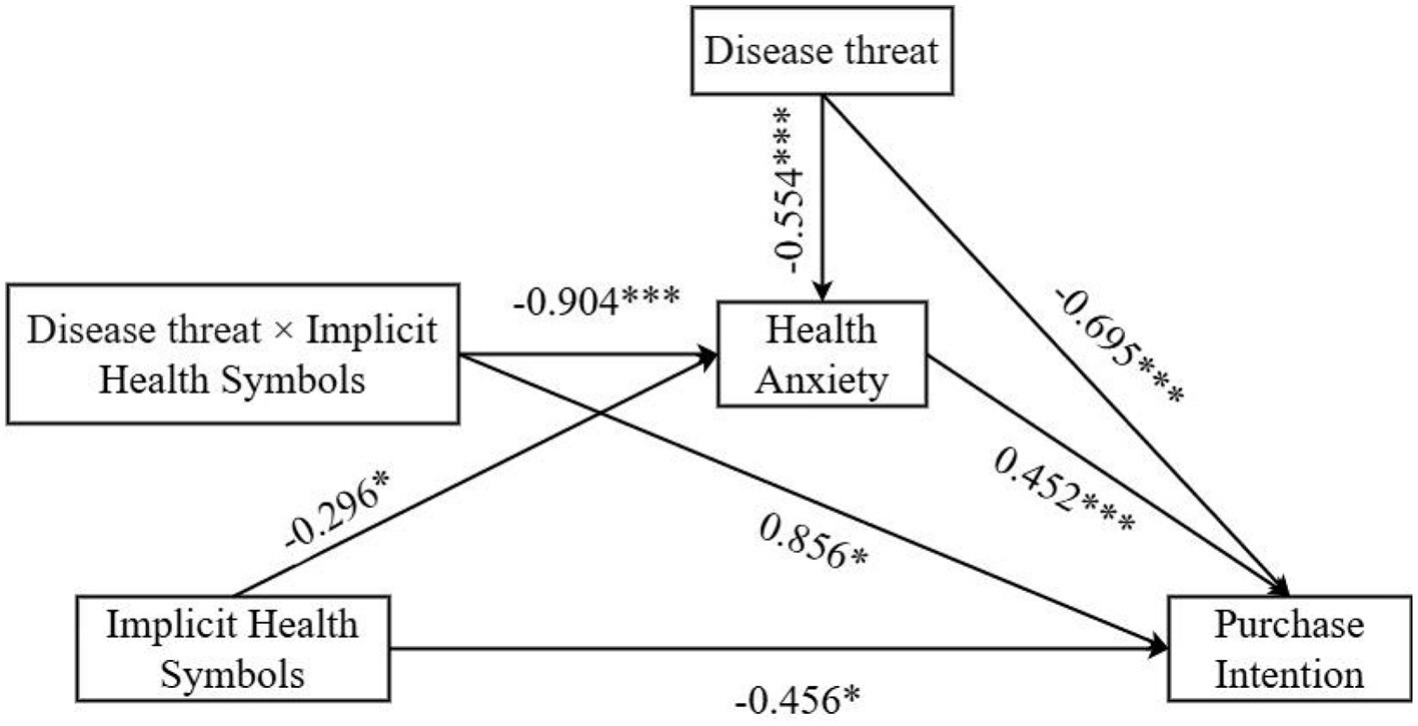
Path coefficient plot for the moderated mediation model. **p < 0.05; ***p < 0.001.

**Figure 5:**
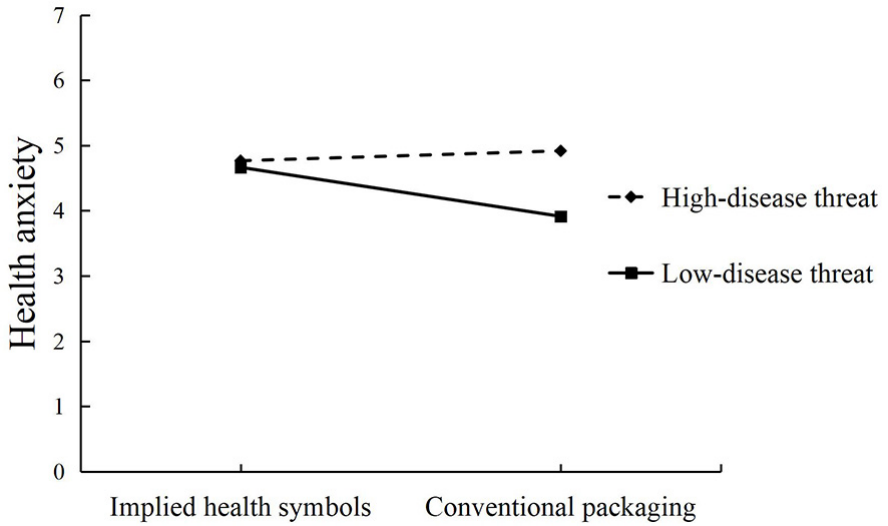
Interaction plot of the effect of the interaction of probiotic products and disease threat on health anxiety.

**Figure 6:**
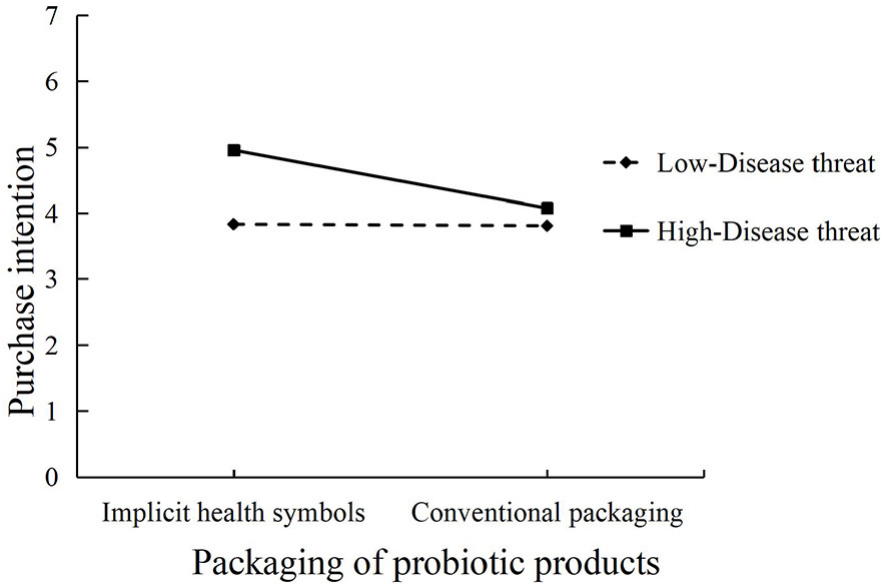
Interaction plot of the effect of the interaction of probiotic products and disease threat on purchase intention.

Moderated mediation was tested using PROCESS Model 8. Implied health symbols significantly predicted purchase intention (β = −0.456, 95% CI = [−0.805, −0.107], *p* = 0.011), disease threat significantly predicted purchase intention (– = −0.695, 95% CI = [−1.052, −0.339], *p* < 0.001), and health anxiety significantly predicted purchase intention (β = 0.452, 95% CI = [0.297, 0.607], *p* < 0.001). Disease threat also significantly predicted health anxiety (β = −0.554, 95% CI = [−0.788, −0.319], *p* < 0.001), and health anxiety significantly predicted purchase intention (β = −0.296, 95% CI = [−0.612, −0.529], *p* = 0.013), as shown in Figure 4. The interaction between implied health symbols and disease threat significantly affected purchase intention (β = 0.856, SE = 0.359, *p* = 0.018, 95% CI = [0.150, 1.563]) and health anxiety (β = −0.904, SE = 0.238, *p* < 0.001, 95% CI = [−1.372, −0.436]), as shown in Figures 5, 6.

Further examination of the conditional effects revealed that when disease threat was high, implied health symbols exerted a significant direct effect on purchase intention (β = −0.879, SE = 0.248, *p* < 0.001, 95% CI = [−1.367, −0.392]), but the indirect effect through health anxiety was not significant (β = 0.068, SE = 0.074, 95% CI = [−0.083, 0.215]). When disease threat was low, the direct effect became non-significant (β = −0.023, SE = 0.257, *p* = 0.928, 95% CI = [−0.529, 0.483]), whereas the indirect effect through health anxiety was significant (β = −0.341, SE = 0.098, 95% CI = [−0.551, −0.168]). The index of moderated mediation was significant (β = −0.409, SE = 0.125, 95% CI = [−0.670, −0.182]). These results supported Hypotheses 3a and 3b.

Because packaging type and disease threat are dummy-coded experimental conditions, the sign of the coefficient depends on the chosen reference category. In the present coding scheme (*X* = 0 implicit-symbol packaging; *X* = 1 conventional packaging), a negative coefficient would mean that implicit-symbol packaging yields higher outcomes than conventional packaging, whereas a positive coefficient would mean lower outcomes. For ease of interpretation, we therefore emphasize cell means; simple effects within each threat condition; plotted interactions (Figures 5, 6), which directly show the direction and magnitude of the effects.

### 6.3 Discussion

The study 3 shows that when perceived disease threat is high, implicit health-symbol framing exerts a comparatively stronger direct effect on purchase intention. Under high threat, individuals are motivationally oriented toward immediate risk management; thus, medical-scientific packaging cues are more likely to be construed as credible, efficacy-implying cues to action that facilitate a “danger-control” response (i.e., adopting an available coping option). In such contexts, purchase intention can be triggered with less dependence on the intermediate affective state of health anxiety, because the decision is driven by urgency and coping appraisal (perceived response efficacy) rather than by extended worry-based inference.

In contrast, under low threat, consumers experience less urgency and greater interpretive latitude. Implicit medical-scientific cues can then operate primarily as ambiguous health-relevant signals that heighten state health anxiety, which subsequently motivates purchase as a reassurance-seeking strategy. This explains why the indirect effect via health anxiety is more prominent under low threat, whereas the high-threat condition exhibits partial “de-mediation,” with the decision path becoming more proximal and behaviorally oriented.

## 7 Discussion

### 7.1 Theoretical implications

This research provides empirical validation of the influence of implicit health symbols on probiotic packaging on the purchase intention of patients with gastrointestinal disorders, contributing to health marketing theory grounded in the Health Belief Model (HBM) (93). Our findings demonstrate that subtle non-verbal packaging cues—such as microbial imagery, intestinal icons, and laboratory-style layouts—function as heuristic cues that amplify perceived benefits and sensitivity to health threats, thereby driving consumer behavior in this population. Unlike prior studies that predominantly examined explicit health claims among general consumers (94), the present work shows that implicit symbols influence behavioral intentions through peripheral processing pathways without requiring deliberative evaluation of propositional content. This finding extends dual-process models of persuasion by demonstrating that implicit packaging cues can achieve behavioral influence comparable to explicit claims, particularly among consumers whose chronic conditions heighten attentional vigilance toward health-related signals. Drawing on converging perspectives from sensory marketing, semiotics, and attribution theory, we propose that implicit health symbols serve as cognitive anchors that stabilize uncertain health narratives: patients with gastrointestinal disorders attribute greater therapeutic value to products bearing such symbols, interpreting them as endorsements of symptom relief rather than mere aesthetic features. This attribution process, combined with the positive affective responses that implicit cues generate—consistent with affect-as-information theory—helps explain why implicit symbols outperform neutral packaging designs in driving purchase intention (H1). Our multi-study design involving 955 participants provides convergent evidence for this effect, complementing the literature on functional food marketing and addressing gaps in prior probiotic consumption research that overlooked disease-specific cohorts.

The identified mediating role of health anxiety provides insight into the psychological pathways linking implicit health symbols to behavioral outcomes. From a cognitive-behavioral perspective, health anxiety serves as a mechanism that translates symbolic exposure into purchasing motivation: implicit health symbols activate threat schemas associated with intestinal dysfunction, which in turn prompt compensatory purchasing as a form of reassurance-seeking. This mediation extends the HBM by illustrating how implicit cues can amplify anxiety through heightened interpretation of bodily signals, channeling arousal into volitional intention—a process consistent with Protection Motivation Theory's conceptualization of anxiety as an adaptive response directing symbol-induced arousal toward self-protective behavior. Our analysis provides evidence for this pathway within a disease-specific context, complementing prior functional food research that has suggested but not rigorously tested anxiety's mediating influence. By demonstrating that health anxiety bridges perceptual and behavioral domains, these results highlight the importance of incorporating emotional mediators into models of health-related consumer behavior.

The moderating role of disease threat identifies the boundary conditions of implicit health symbols' influence. Our interaction analyses reveal that high perceived disease threat amplifies both the direct effect on purchase intention and the mediated effect through health anxiety, positioning threat as a contextual enhancer of symbol processing. This finding extends the HBM by clarifying how threat perception escalates perceived susceptibility, directing cognitive resources toward implicit health symbols as coping anchors under high-threat conditions while attenuating their influence under low-threat scenarios. The moderated mediation analysis further reveals a qualitative shift in the processing pathway: under high threat, the effect operates more directly through action-oriented coping appraisal, whereas under low threat, the indirect pathway through health anxiety is more prominent. These results contribute to understanding contextual vulnerability in functional nutrition and support models of consumer health behavior in which situational moderators demarcate meaningful boundary conditions, with relevance for marketing communication during periods of elevated public health concern.

Health-related psychological arousal in response to packaging cues spans a qualitative continuum from adaptive concern to maladaptive anxiety (95, 96). Adaptive health concern denotes a proportionate attentiveness to health-relevant information that supports informed decision-making, whereas maladaptive health anxiety involves disproportionate, ruminative worry that exceeds what is warranted by one's actual health status (94). Our operationalization of health anxiety as a mediating variable likely captures variance from both ends of this continuum, and our data do not permit a clean separation of the two components. This limitation is consequential because the ethical and practical implications diverge depending on which component predominates: if implicit health symbols primarily activate adaptive concern, the mechanism is informationally valuable; if they primarily activate maladaptive anxiety, the mechanism is ethically problematic. Future research should address this ambiguity through measurement approaches that distinguish between these components and by experimentally manipulating the congruence between implicit health symbols and actual product health attributes.

An important conceptual issue raised by our findings concerns the boundary between implicit health symbols and explicit health claims. We recognize that this boundary is more accurately characterized as a continuum than as a binary distinction (61). At one end lie overt, propositional health claims that are subject to regulatory scrutiny in most jurisdictions; at the other lie purely aesthetic design choices with no discernible health connotation. The implicit symbols examined in the present study occupy an intermediate zone—they do not assert a specific health benefit in propositional terms, yet they are sufficiently imbued with health-related meaning to activate health anxiety and influence purchase intentions, as our data demonstrate. This intermediate positioning is theoretically significant because it suggests that the persuasive influence of packaging on health-related consumer behavior does not require explicit claims; rather, symbolic and associative cues operating below the threshold of propositional content can achieve comparable psychological effects. This finding aligns with dual-process models of persuasion (45), which hold that peripheral or heuristic cues can drive attitudes and behavior with minimal conscious deliberation—a process that may be especially pronounced among health-anxious individuals who are motivated to attend to any signal of relevance to their condition.

The conceptual ambiguity of implicit health symbols carries direct and consequential implications for regulatory policy. Current food labeling regulations in most jurisdictions focus predominantly on explicit, text-based health and nutrition claims, requiring that such claims be scientifically substantiated and pre-authorized. However, these frameworks generally do not extend to non-verbal symbolic elements such as imagery, color associations, or graphic motifs that implicitly suggest health benefits without making a falsifiable assertion. Our findings suggest that this regulatory gap may be consequential: implicit symbols can produce measurable shifts in health anxiety and purchase intention that are functionally similar to the effects one might expect from explicit claims, yet they remain largely invisible to existing regulatory mechanisms. This observation echoes concerns raised by scholars of food marketing regulation, who have argued that the narrow focus on propositional claims allows manufacturers to communicate health-related messages “by other means.” We therefore recommend that regulatory bodies consider adopting a broader, effects-based approach to health communication oversight—one that evaluates packaging elements not solely by their propositional content but by their demonstrated capacity to influence health-related cognitions and behaviors in consumer populations, particularly vulnerable groups such as patients with chronic conditions.

### 7.2 Practical implications

The present findings have practical implications for stakeholders involved in probiotic product communication and labeling, particularly in contexts where consumers with gastrointestinal disorders may be considered a health-vulnerable population. While our experiments show that implicit health-symbol framing can increase purchase intention, this empirical regularity should be interpreted primarily as a consumer-protection and governance signal rather than as a prescriptive guide for persuasion.

First, for manufacturers and brand owners, the results underscore the need to treat medical-scientific aesthetic cues (e.g., microbiome-like iconography, cool “clinical” color palettes, and technical-looking identifiers) as high-responsibility design elements because they can shape behavior through heightened health-related affect. Accordingly, packaging should be designed to reduce inference-based ambiguity and support informed evaluation: provide clear strain identity, viable counts (e.g., at end of shelf-life where applicable), intended-use boundaries, and plain-language explanations of what is and is not supported by evidence. Where visual elements are used to facilitate comprehension, they should be evidence-congruent and accompanied by accessible substantiation (e.g., QR codes linking to peer-reviewed summaries or third-party evidence reviews). Digital tools that accompany packaging (e.g., educational apps) should prioritize health literacy and decision support, rather than intensifying urgency or threat.

Second, the identified mediating role of health anxiety indicates that implicit cues may inadvertently increase emotional distress and motivate purchasing as reassurance seeking. This highlights an ethical requirement for transparency and emotional safety: industry associations and firms should adopt guidance that discourages designs likely to induce undue worry, and should implement pre-release testing that includes not only perceived efficacy and purchase intention but also state anxiety, perceived manipulation, and anticipated regret among patient groups. These measures help ensure that packaging effects, when they occur, reflect adaptive health concern and informed choice rather than distress-driven consumption.

Third, the moderating role of disease threat suggests that the influence of implicit health-symbol framing can be stronger in heightened threat contexts. This result should not be construed as a rationale to intensify health-suggestive cues during outbreaks or other high-threat periods. On the contrary, it supports a heightened duty of care and a context-sensitivity constraint: stakeholders should avoid coupling medical-scientific aesthetics with threat-salient messaging environments and should use additional safeguards (e.g., clearer disclaimers, stronger evidence transparency, and stricter review of implied meanings) when public concern is elevated.

Responsible marketing considerations. Although implicit health symbols may increase purchase intention, our mechanism indicates that part of this effect can occur via elevated health anxiety. Therefore, packaging optimization should not be pursued as a strategy to “leverage anxiety” among patients. Instead, firms should adopt a responsibility-by-design approach that prioritizes informed choice: provide strain- and dose-specific information, evidence links, clear disclaimers that packaging symbols are illustrative rather than therapeutic guarantees, and avoid fear-priming narratives or outbreak-themed promotional tactics. This approach reduces the risk that marketing success is achieved through emotional coercion rather than genuine perceived benefit.

From a policy perspective, the amplified influence of implicit health-symbol framing under high disease-threat conditions provides grounds for effects-based regulatory attention aimed at protecting vulnerable consumers from misleading overall impressions. Agencies such as the U.S. Federal Trade Commission (FTC) or the European Food Safety Authority (EFSA) could consider guidance that extends beyond propositional claims to the net impression conveyed by integrated design ensembles, and may require substantiation or modification where packaging is reasonably likely to imply treatment-like efficacy. Policies could also encourage or mandate standardized disclosures (e.g., “Symbols are illustrative; consult a clinician for personalized advice”) and promote evidence-access mechanisms (e.g., QR-linked substantiation) to counter overreliance on visuals. International bodies (e.g., Codex Alimentarius) could incorporate guidance on implied claims to support cross-border harmonization, while public health initiatives can improve “symbol literacy” to help patients interpret packaging cues in an evidence-calibrated manner.

In clinical practice, the findings can inform patient education and shared decision-making. Healthcare professionals (e.g., gastroenterologists and dietitians) may proactively address how packaging cues influence expectations and anxiety, guide patients toward evidence-supported probiotic options, and discourage substitution of probiotics for indicated medical care. Collaborative initiatives among clinics, researchers, and responsible manufacturers could develop patient-facing evidence summaries for common strains and indications, helping patients translate packaging impressions into informed, low-anxiety choices. Overall, these implications support a governance-oriented ecosystem in which improved transparency and ethical restraint allow beneficial products to be communicated responsibly while reducing the risk of anxiety-driven consumption.

### 7.3 Ethical implications and risks of exploitative marketing

Our findings demonstrate that implicit symbols on food packaging can elevate health anxiety among patients with gastrointestinal diseases, and that this heightened anxiety subsequently drives purchase intention. While this mechanism is presented as an empirical observation aimed at advancing scientific understanding, we acknowledge that it raises significant ethical concerns that warrant explicit discussion.

Patients with gastrointestinal diseases constitute a vulnerable consumer group in several respects. Recurrent and unpredictable symptoms heighten attentional bias toward health-related cues and increase reliance on heuristic processing under uncertainty. Information asymmetry is pronounced in the probiotic market—strain heterogeneity, dosage variability, and inconsistent clinical evidence limit patients' ability to independently evaluate implied efficacy. Furthermore, anxiety can narrow cognitive scope and shift decisions from deliberation to reassurance-seeking. These vulnerability dimensions raise a fundamental concern about informed autonomy: whether purchase intentions formed through anxiety-mediated pathways reflect free and informed consumer choice, or whether they more closely resemble a form of emotional influence that compromises decision-making agency. The ethical boundary between acceptable persuasion and exploitation is particularly salient in this context. Packaging elements that implicitly suggest medical-grade efficacy may function as “quasi-claims” that shape therapeutic expectations without making falsifiable statements. When such cues are designed or deployed to capitalize on patients' fear of deterioration—especially during high-threat contexts—they risk becoming exploitative by amplifying worry beyond what is clinically warranted, encouraging over-purchasing as reassurance behavior, and potentially displacing evidence-based care. The resulting harm extends beyond financial loss to include emotional distress, regret, and reinforcement of maladaptive safety-seeking cycles. We therefore emphasize that the identification of this pathway should serve as evidence calling for greater vigilance and ethical scrutiny in health-related commercial communication, rather than as an actionable marketing strategy.

From a regulatory perspective, our operationalization treats packaging elements as “implicit” when they do not contain a direct efficacy proposition or disease endpoint claim and instead function as associative cues that invite inference. This approach is consistent with the claim-based focus of prevailing food-labeling regulations, while also reflecting the recognized regulatory gray zone surrounding implied claims conveyed through visual symbolism and overall impression. Current regulatory frameworks predominantly target explicit, propositional health claims, leaving a gap for non-verbal symbolic cues that can nevertheless produce measurable changes in anxiety and intention. Our results support an effects-based perspective on oversight: packaging should be evaluated not only by whether it contains explicit health claims, but also by whether its overall design is reasonably likely to imply therapeutic efficacy to vulnerable audiences and to induce undue anxiety. From an industry standpoint, responsible design should prioritize accuracy and consumer welfare by aligning health-suggestive imagery with substantiated evidence, avoiding fear-evoking motifs, providing accessible evidence transparency tools, and exercising heightened caution in targeted advertising to patient communities and in high-threat messaging environments.

Non-prescriptive clarification. The present research is descriptive and explanatory. It should not be construed as recommending the use of anxiety-eliciting cues to increase sales; rather, it highlights a vulnerability pathway that warrants ethical safeguards, transparency, and appropriate regulatory attention.

### 7.4 Adaptive health concern vs. maladaptive anxiety exploitation

Health-related arousal elicited by packaging cues is not psychologically homogeneous. We therefore delineate adaptive health concern from maladaptive anxiety exploitation to clarify the ethical meaning of our findings in vulnerable patient groups.

Adaptive health concern refers to a proportionate, reality-based increase in vigilance that motivates information-seeking and deliberative evaluation (e.g., checking strain identity, dose, contraindications, and evidence) and is congruent with substantiated product attributes. In this case, packaging functions as a cue to action that supports informed self-management without impairing autonomy. Maladaptive anxiety exploitation, in contrast, refers to the use of health-suggestive aesthetics and contextual cues in ways that disproportionately amplify perceived threat, trigger catastrophic interpretations of symptoms, or promote reassurance-seeking purchasing that is weakly connected to evidence of benefit. This pattern is ethically concerning because it can undermine informed choice, increase distress, and reinforce safety behaviors that are clinically characteristic of health anxiety.

Practically, the boundary between these two states can be evaluated along three auditable dimensions: (1) evidence congruence (whether the implied medical-scientific impression matches strain-/dose-specific evidence and appropriate indications), (2) autonomy and transparency (whether consumers are provided accessible, verifiable information rather than relying on implication), and (3) psychological and behavioral outcomes (constructive vigilance vs. distress-driven, repetitive purchasing). Our empirical model identifies an anxiety-mediated pathway as an observed mechanism; it does not imply that anxiety should be elicited as a design objective.

### 7.5 A responsibility framework for marketing to vulnerable populations

Because gastrointestinal disease patients constitute a health-vulnerable consumer segment, the application (or regulation) of implicit health-symbol framing should be guided by a responsibility-by-design approach. We propose four normative principles that translate our empirical findings into ethically constrained practice and oversight.

Truthfulness-by-design. Any health-suggestive aesthetic (e.g., “clinical” layouts, strain-like identifiers, micrograph-like imagery) should be aligned with verifiable product information (strain identification, viable counts at end of shelf-life, recommended use, and limitations of evidence). Where evidence is mixed or indication-specific, packaging should avoid an undifferentiated “medical-grade efficacy” impression.

Non-maleficence and emotional safety. Designs and messages should be screened for undue fear arousal among patient groups, especially under elevated threat contexts. A practical implementation is to incorporate pre-market user testing that measures not only perceived efficacy and purchase intention but also state anxiety, perceived manipulation, and regret.

Transparency and autonomy preservation. To reduce reliance on inference, packaging should provide accessible routes to substantiation (e.g., QR codes linking to plain-language evidence summaries, strain-specific references, and guidance to consult clinicians for individualized decisions). Transparency is particularly important because implicit cues operate below the threshold of propositional claims yet may exert comparable behavioral influence.

Targeting restraint and context sensitivity. Firms and regulators should exercise heightened caution in targeted marketing to patient communities and avoid coupling medical-scientific aesthetics with threat-amplifying contexts (e.g., outbreak-themed promotions). Oversight can adopt an effects-based lens: whether the overall impression is reasonably likely to imply treatment-like efficacy to vulnerable audiences and whether it elevates distress beyond what is warranted.

These principles are intended to prevent a slippage from legitimate communication into ethically problematic influence, while preserving the possibility that well-substantiated probiotics can be communicated responsibly to patients who may benefit.

### 7.6 Cultural particularity and external adaptability

The present findings were obtained exclusively within a Chinese consumer sample, and several features of the Chinese cultural context may have amplified the effects we observe. Most fundamentally, Chinese culture is characterized by a long-standing tradition of food–medicine homology, in which food and medicine are understood as occupying a continuum rather than as categorically distinct domains (97, 98). This cultural schema provides a pre-existing cognitive infrastructure for interpreting food packaging as health-relevant, potentially rendering Chinese consumers more receptive to implicit health symbols than consumers in cultures that maintain a sharper boundary between food and pharmaceutical products. Additionally, China is widely characterized as a high-context culture (99) in which meaning is routinely conveyed through implication, contextual cues, and nonverbal signals (100). Consumers socialized in this communicative tradition may possess heightened sensitivity to implicit packaging cues—an interpretive skill that would amplify the pathway from implicit health symbols to health-related cognition. Finally, the Chinese food market's complex regulatory environment, in which the boundaries among ordinary foods, health foods, and quasi-pharmaceutical products are frequently blurred in marketing practice (101), may have cultivated consumer interpretive habits that differ from those in markets with stricter and more consistently enforced health claim regulations, such as the post-2006 European Union market. These three cultural factors—philosophical tradition, communicative norms, and regulatory environment—constitute important boundary conditions on the generalizability of our findings.

We do not expect that the core psychological mechanism identified in this study—the activation of health anxiety by health-suggestive packaging cues—is unique to Chinese consumers. Research conducted in Western contexts has consistently demonstrated that visual packaging elements such as green coloring, natural imagery, and organic-suggestive design influence health perceptions and product evaluations among European and North American consumers (102–104). Health anxiety, similarly, is a well-documented psychological construct across cultures (95, 96). We therefore expect the existence of the implicit health symbol → health anxiety → purchase intention pathway to be cross-culturally robust. However, we predict that the magnitude of the effect would be moderated by at least three cultural factors. First, consumers in low-context cultures (99) may require more explicit health cues to generate equivalent levels of health inference, potentially attenuating the effect of purely implicit symbols while preserving or enhancing the effect of quasi-explicit textual elements. Second, the specific visual and textual symbols that carry implicit health connotations are partly culturally constructed: imagery rooted in Traditional Chinese Medicine may not carry equivalent health associations for Western consumers, just as Western “clean label” aesthetics may not fully transfer to Chinese markets. Third, cross-cultural differences in regulatory literacy and consumer skepticism toward marketing claims may moderate the downstream effects on purchase intention. European consumers, who are exposed to the EU's comparatively stringent regulation of health claims, may have developed more critical interpretive frameworks that attenuate the persuasive impact of implicit health cues. Empirically disentangling these cultural moderators requires cross-national experimental designs with culturally adapted stimuli—a promising direction for future research.

A further consideration concerns our reliance on self-reported gastrointestinal disease status to classify participants into health-status groups. This approach introduces potential misclassification: some participants who reported having a gastrointestinal condition may not have a clinically confirmed diagnosis, while others with undiagnosed conditions may have been classified as healthy. Such measurement imprecision would generally be expected to introduce noise that attenuates between-group differences, making our observed effects a conservative estimate. More importantly, however, we argue that self-reported health status may be the more theoretically appropriate operationalization for the present research, given that our proposed mechanism operates through health anxiety—a psychological state driven by perceived health vulnerability rather than by objective clinical status. The Health Belief Model (105), Protection Motivation Theory (106), and the Extended Parallel Process Model (107) converge on the principle that perceived susceptibility, rather than objective risk, is the primary determinant of health-related behavioral responses. A consumer who perceives themselves as having a gastrointestinal condition is, from the perspective of our model, the consumer whose health anxiety is most likely to be activated by health-suggestive packaging—regardless of whether that perception has been clinically validated. Nevertheless, we acknowledge that the absence of clinical verification limits our ability to determine whether the observed effects generalize to consumers with formally diagnosed conditions, whose illness severity, medical literacy, and treatment status may differ systematically from those of self-diagnosed individuals. Future research could address this limitation by incorporating validated screening instruments (e.g., the Rome IV criteria for functional gastrointestinal disorders) (53) alongside self-report measures, thereby enabling a direct comparison of effects across subjectively and objectively defined health-status groups.

### 7.7 Limitations and directions for future research

Although the present research provides robust empirical evidence through three rigorously designed experiments involving a large sample of gastrointestinal disease patients, several limitations must be acknowledged, which simultaneously open avenues for future investigation.

First, the identification of participants as patients with gastrointestinal disease relied on self-reported diagnosis through an online survey platform, without independent clinical verification such as medical record review, endoscopic examination, or laboratory confirmation. Although participants were asked to specify the type of gastrointestinal disease and to confirm that the diagnosis had been made by a healthcare professional, the possibility of misclassification—including both the inclusion of individuals without true gastrointestinal conditions and the exclusion of undiagnosed patients—cannot be ruled out. This may limit the external validity and generalizability of our findings to clinically confirmed gastrointestinal patient populations. Self-reported diagnosis is a widely used approach in online survey-based research due to practical constraints; however, the inherent limitations of this method should be considered when interpreting the results. Future studies are encouraged to recruit participants from clinical settings with verified diagnoses, or to cross-validate self-reported data with electronic health records, in order to enhance diagnostic accuracy and strengthen the robustness of the conclusions.

Second, the experimental stimuli, although professionally designed and pre-tested, relied on static two-dimensional packaging images rather than physical products or real retail environments. This approach, while standard in controlled experimentation, may underestimate the multimodal influence of tactile, olfactory, or shelf-context cues that characterize actual purchase scenarios. Moreover, the implied health symbols were operationalized using cool-toned, laboratory-report-style designs emphasizing scientific authority, which may not fully capture the diversity of implicit symbolic strategies employed globally (e.g., nature-inspired warm tones or minimalist Scandinavian aesthetics). Future research could adopt virtual reality or augmented-reality paradigms to simulate realistic shopping experiences and systematically vary multiple symbolic dimensions (e.g., color temperature, iconicity, textual implicitness) in a factorial manner to delineate their interactive effects.

Third, and relatedly, a notable feature of our experimental design is that the implicit health symbols condition operationalizes the theoretical construct as a composite medical-scientific aesthetic framing—an integrated packaging gestalt comprising multiple co-occurring design dimensions (iconographic, chromatic, typographic, technical-lexical, and textual elements). While we have provided theoretical justification for treating these elements as constituents of a single higher-order semiotic construct, this composite operationalization necessarily means that the present design cannot disentangle the relative contributions of individual design dimensions to the observed effects on health anxiety and purchase intention. It remains an open question whether certain elements (e.g., bacterial micrographs or strain identifiers) carry disproportionate weight in driving the medical-scientific aesthetic impression and its downstream psychological consequences, or whether the effect is genuinely holistic and non-decomposable. Future research could address this question through factorial designs that systematically vary individual design dimensions (e.g., iconography × color scheme × typography), thereby enabling an assessment of both main effects and interaction effects among constituent elements. Such designs would clarify whether the persuasive impact of medical-scientific aesthetic framing is best characterized as additive, synergistic, or driven by a single dominant element—information that would carry important implications for both packaging design practice and regulatory oversight.

A further limitation concerns the intensity and content of our disease-threat prime in Study 3. Although the manipulation was designed to increase perceived disease threat, outbreak-related vignettes may also partially activate acute threat processing, panic-related affect, or even mortality-salience/existential threat associations, which could inflate the observed moderation effects. To mitigate this concern, we revised the vignette to minimize explicit death-related content; however, we cannot fully rule out residual overlap between disease-threat appraisal and broader existential threat responses. Future research should (1) employ alternative high-threat primes that are high in perceived severity/susceptibility yet low in mortality salience (e.g., chronic symptom recurrence, flare-up likelihood, or medically framed risk without fatality cues), (2) measure and statistically control mortality salience or death-related thought accessibility, and (3) compare multiple threat manipulations to establish convergent validity for perceived disease threat as the moderator.

Third, although health anxiety was identified as a significant mediator and disease threat as a moderator, the underlying neurobiological and physiological mechanisms remain unexplored. Emerging evidence from psychoneuroimmunology and gut-brain axis research suggests that implied health symbols may modulate vagal tone, hypothalamic-pituitary-adrenal axis activity, or even transient shifts in gut microbiota composition via stress-related pathways. Future studies could incorporate psychophysiological measures (e.g., heart-rate variability, salivary cortisol, fecal calprotectin) and functional neuroimaging (e.g., fMRI focusing on insula and anterior cingulate cortex activation) to elucidate how symbolic exposure translates into visceral and neural responses, thereby bridging psychological and biological levels of analysis.

A methodological limitation is that our scenarios embedded a physician/medical-context recommendation, which may have elevated baseline perceptions of product legitimacy and category appropriateness. This context could plausibly inflate the incremental impact of medical-scientific packaging cues by priming a health-focused interpretive frame and increasing reliance on authority-consistent heuristics. Although the recommendation content was held constant across conditions (supporting internal validity of the packaging manipulation), the findings may generalize more strongly to settings where consumers encounter probiotics in medically salient contexts (e.g., after consultations) than to purely self-initiated retail browsing. Future research should replicate the effects in designs that (a) remove physician-context recommendations entirely, (b) experimentally manipulate recommendation presence/strength (none vs. general suggestion vs. explicit physician endorsement), and (c) test interaction patterns between recommendation cues and packaging cues in more naturalistic shopping environments.

Fourth, the present investigation focused exclusively on purchase intention as the primary outcome, a well-established proximal predictor of behavior. However, the critical question of whether implied health symbols ultimately influence actual consumption, long-term adherence, or clinical outcomes (e.g., symptom severity, quality of life, microbiota diversity) remains unanswered. Longitudinal field experiments or randomized controlled trials partnering with probiotic manufacturers could track real-world purchasing behavior, product usage patterns, and objective health markers over extended periods (e.g., 3–12 months), providing definitive evidence of downstream clinical and economic impact.

Fifth, cultural and regulatory contexts may significantly moderate the effectiveness of implied health symbols. The current study was conducted in China, where implicit health suggestions on food packaging face relatively permissive regulatory oversight compared to the European Union's stringent Regulation (EC) No 1924/2006 or the U.S. FDA's qualified health claim framework. Cross-cultural replications in regions with differing regulatory stringency and collectivist–individualist orientations are warranted to test the boundary conditions of the observed effects and inform globally applicable marketing guidelines.

Finally, ethical considerations merit greater attention in future research. While implied health symbols demonstrably enhance purchase intention among vulnerable patients, their capacity to transiently elevate health anxiety raises concerns regarding potential exploitation of illness-related fears. Experimental designs incorporating explicit measures of post-exposure emotional distress, regret, or perceived manipulation, as well as qualitative follow-up interviews, could illuminate the fine line between persuasive efficacy and psychological harm. Such evidence is essential for developing ethically grounded industry standards and regulatory policies that protect consumer welfare without stifling innovation.

A limitation of our experiments is that the packaging manipulation employed a deliberately stylised, high-contrast difference between conditions to strengthen causal identification of implicit health-symbol framing. While this improves experimental control, it may reduce ecological validity because real-world probiotic packages often differ more subtly and combine “medical–scientific” cues with mainstream commercial aesthetics. Consequently, the observed effect sizes may be inflated relative to typical marketplace exposures, and generalization to natural purchase environments should be made cautiously. Future research should therefore test the model using field experiments or naturalistic online shopping simulations with competing brands and realistic choice sets; real products or professionally photographed packages with minimal artificial contrast.

In conclusion, although the present multi-study investigation establishes implied health symbols as a potent driver of probiotic consumption among gastrointestinal disease patients through anxiety-mediated and threat-moderated pathways, the limitations outlined above highlight fertile directions for future scholarship. By addressing sampling representativeness, ecological validity, neurobiological mechanisms, behavioral and clinical outcomes, cross-cultural generalizability, and ethical implications, subsequent research can further solidify the theoretical edifice of health marketing while translating empirical insights into responsible, evidence-based practices that ultimately benefit both patients and public health.

## Data Availability

The raw data supporting the conclusions of this article will be made available by the authors, without undue reservation.
