# Supplementary material for: The impact of implicit health symbols on probiotic products on purchase intention among gastrointestinal disease patients: evidence from multiple experiments
Source: Front Nutr. 2026 Mar 27;13:1751124. doi: 10.3389/fnut.2026.1751124 (PMC13065513; doi:10.3389/fnut.2026.1751124)
Supplement: Supplementary file 1 [file Table_1.docx]

# The Impact of Implicit Health Symbols on Probiotic Products on Purchase Intention Among Gastrointestinal Disease Patients: Evidence from Multiple Experiments

# Experiment 1

Dear friend, hello, please imagine that you recently went to the hospital for treatment due to unbearable diarrhea. After your doctor's treatment, you were diagnosed with antibiotic-associated diarrhea. These diseases are caused by antibiotics disrupting the balance of the intestinal flora, leading to diarrhoea. Doctors suggest that you can go to the pharmacy to buy probiotic products for appropriate supplementation. You are now buying the right probiotic product at a large pharmacy near you.

# Experiment 2

Dear friends, please imagine that you recently feel bloating, indigestion, and occasionally constipation due to long-term work stress and irregular diet. You go to the department of Gastroenterology of the hospital. After consultation and examination by the doctor, you are diagnosed with functional gastrointestinal disorder, and the doctor points out that this may be related to intestinal flora imbalance. After prescribing some drugs to relieve symptoms, the doctor specifically suggests that you can try to supplement some probiotics to help regulate intestinal function and restore internal balance. You are now in the chain drugstore downstairs, choosing the right probiotic product according to your doctor's advice.

# Experiment 3

Dear friend, imagine that you are about to travel to a region where health conditions may differ from those at home. You are concerned that you may have traveler's diarrhea and go to the travel clinic of your hospital for consultation. After giving you regular travel health advice, your doctor tells you that studies have shown that taking certain probiotic strains (such as certain Lactobacillus and Bifidobacterium) in advance can help strengthen your gut and help prevent traveler's diarrhea. Doctors recommend that you start taking it 1-2 weeks before departure and continue to replenish it for the duration of your trip. You are now at the pharmacy counter of a large supermarket, carefully comparing the ingredient lists of different probiotic products, hoping to find the kind of preventive strain mentioned by the doctor.

## High disease threat stimuli

Recently, an outbreak of **cholera** caused by Vibrio cholerae has been reported in multiple countries and regions, with case numbers increasing in some areas. Health authorities have issued high-level alerts and reminded the public to stay vigilant.

The pathogen can spread through contaminated water and food and is highly contagious. After infection, V. cholerae proliferates in the intestine and produces toxins, which may cause sudden onset of **profuse watery diarrhea** and **severe vomiting**, sometimes within hours. Because fluid can be lost rapidly, patients may quickly develop **dehydration**, **electrolyte disturbances**, and **metabolic acidosis**. Typical symptoms can include dry skin, deep-set eyes, hoarseness, a noticeable drop in blood pressure, and a weak pulse. **Timely medical care is critical**, and health agencies emphasize early recognition and prompt treatment to reduce complications.

At present, the outbreak is still developing, reminding the public that the threat of gastrointestinal infectious disease can be **real, close, and unpredictable**. People are advised to pay close attention to hygiene and safe drinking water, and to seek medical advice promptly if severe gastrointestinal symptoms occur.

## Low disease threat stimuli

According to a recent monitoring report by the environmental protection department, the domestic water supply system in some areas of our city has been lightly affected due to an accidental pipeline rupture and leakage, resulting in water quality that has not yet reached the daily optimal standard.

The affected water may contain small amounts of common environmental microorganisms such as Escherichia coli. A small number of individuals with reduced immunity, such as young children, the elderly, or those with underlying medical conditions, may experience **mild gastrointestinal discomfort** after drinking or using this source of water. Possible symptoms include occasional abdominal discomfort, minor changes in bowel habits, or transient loss of appetite. In the vast majority of healthy adults, the body can cope on its own and may not produce any noticeable symptoms.

Relevant departments have quickly intervened and are carrying out pipeline repairs and enhanced disinfection of the water supply system. This incident has been characterized as a local and controllable public environmental health issue. Residents are advised to follow the recommended household water practices (e.g., boiling water for drinking) during this period to **substantially reduce** potential risk. At present, there are no reports of clusters of illness associated with this fluctuation in water quality, and the overall public health risk is considered low.

# Experiment S1：A stricter operationalization of implicit health symbols

Method

**Design and participants.** Study S1 employed a single-factor between-subjects design (packaging: strict-implicit symbols vs. conventional packaging) among self-reported gastrointestinal disease patients recruited via Credamo. Participants were randomly assigned to one of the two conditions.

**Stimuli.** To ensure that the manipulation reflected **implicit symbols rather than explicit health claims**, all claim-like or outcome-propositional phrases (e.g., “clinically verified,” “repair,” or direct statements of therapeutic efficacy) were removed. The strict-implicit condition relied exclusively on non-propositional cues such as microbiome/gut-related iconography, cool color schemes, laboratory-report-like typography/layout, and strain identifiers, whereas the control condition used conventional commercial design elements without medical/scientific aesthetics.

**Measures.** In addition to purchase intention, we included manipulation checks that separately assessed (1) perceived **claim explicitness** (e.g., “The package makes explicit health or medical claims”) and (2) perceived **implicit health suggestion/medical–scientific impression** (e.g., “The package suggests health benefits without stating them explicitly”), both on 7-point Likert scales.

Result

**Manipulation checks.** Participants in the strict-implicit condition perceived a stronger medical–scientific impression / implicit health suggestion (M = 5.06, SD =1.531) than those in the conventional condition (M = 4.21, SD = 1.993, t = -3.620, p < 0.001), while perceived claim explicitness did not increase (t = -1.540, p = 0.125), indicating that the manipulation strengthened implied health signaling without being interpreted as explicit claim-making.

**Main effect on purchase intention.** Replicating the main findings, purchase intention was higher in the strict-implicit condition (M = 5.87, SD = 1.532) than in the conventional condition (M = 5.17, SD = 1.817; F(1, 226) = 9.682, p = 0.002, η2= 0.041).

# Material of stimulation


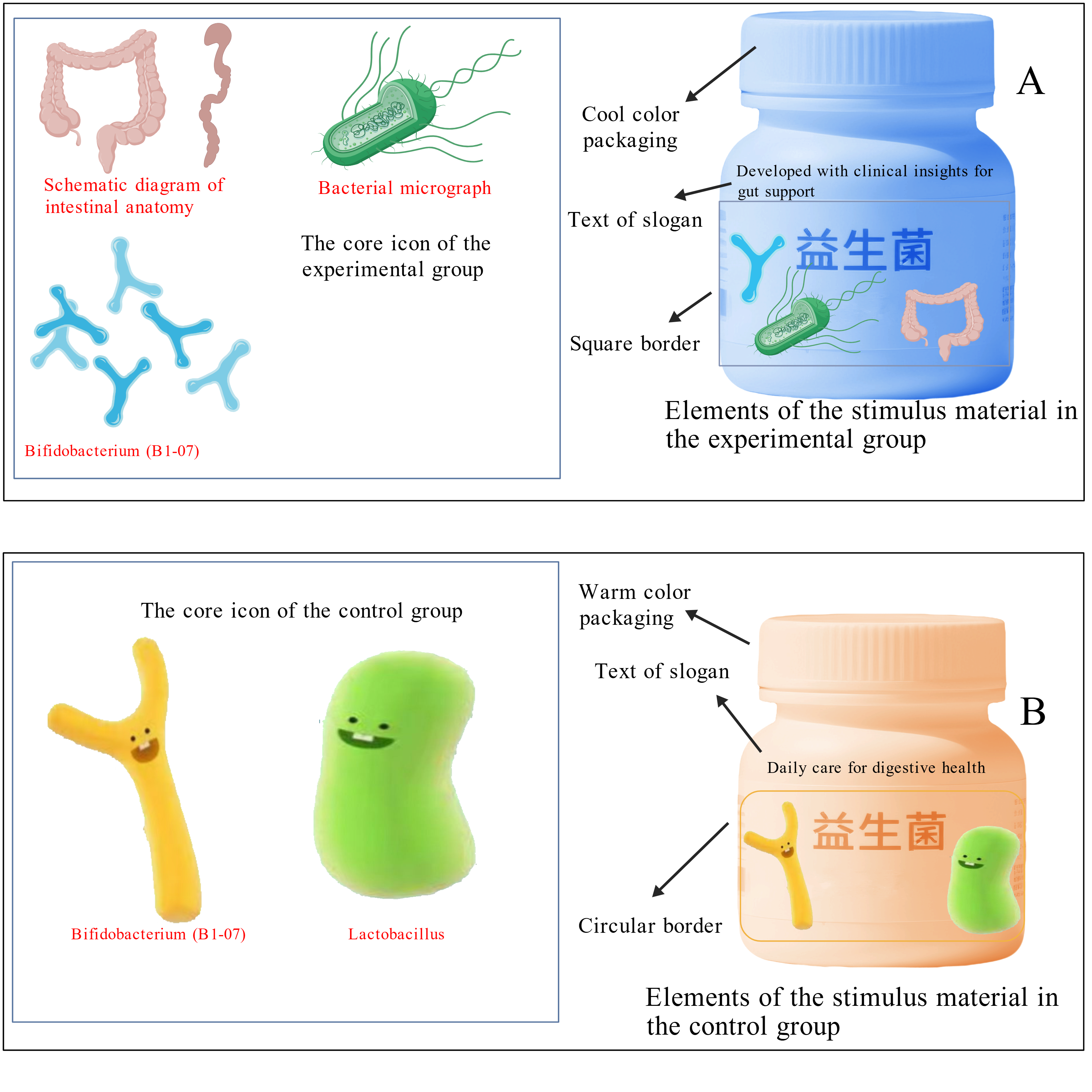


The experimental group’s implicit health symbols in this study leverage the visual cognition system by employing cold color schemes proven to activate associations with “calmness,” “professionalism,” and “technology,” thereby enhancing perceived credibility and efficacy [1]. Microscopic bacterial images and gut cross-sectional diagrams serve as indexical signs pointing to the microbial world and the body’s internal mechanisms, conveying authority [2]. This visual rhetoric, combined with a laboratory report-style layout, guides consumers through a systematic processing akin to reading scientific literature [3]. In contrast, the control group’s warm color schemes evoke associations with “naturalness,” “vitality,” and “gentleness,” while their abstract microbial and plant illustrations function as symbolic signs conveying vague health associations, relying on low-effort heuristic processing [4]. At the level of professional signage, the specific strain designation “Lactobacillus ABC-1” serves as a precise scientific code communicating research depth and standardized production processes [5], while the generalized claim “contains live probiotics” offers limited informational value and fails to support differentiated brand trust.

The textual language and overall layout of the packaging collectively construct the product’s core narrative framework, profoundly shaping consumers’ interpretation of the product’s meaning [6, 7]. This textual information aligns synergistically with the aforementioned visual symbols, reinforcing the narrative of “evidence-based medical intervention” [8]. The laboratory report layout further solidifies this narrative through structured, data-driven visual language that suggests precise measurement and rigorous conclusions, lowering consumers’ thresholds for skepticism. In contrast, the control group’s slogan, “daily digestive health care,” adopts a gentle “everyday wellness maintenance” narrative, emphasizing safety, universality, and lifestyle integration. The minimalist, commercial design aligns with fast-moving consumer goods aesthetics, prioritizing usability and affability but diminishing the perception of professional authority. This narrative difference fundamentally reflects differing target consumer needs: the experimental design specifically targets gastrointestinal disease patients with clear pathological discomfort seeking effective solutions, emphasizing “efficacy” and “evidence,” while the control design caters to health-conscious general consumers seeking daily wellness maintenance, focusing on “safety,” “gentleness,” and “lifestyle integration.”

1. Labrecque LI, Patrick VM, Milne GR. The marketers’ prismatic palette: A review of color research and future directions. Psychology & Marketing. 2013;30(2):187-202.

2. Pauwels L. Visual cultures of science: rethinking representational practices in knowledge building and science communication: UPNE; 2006.

3. Mengis J, Eppler MJ. Wissensdialoge: dimensionen, prinzipien und probleme der gesprächsbasierten wissenskommunikation. Wissenskommunikation in Organisationen: Methoden· Instrumente· Theorien: Springer; 2004. p. 88-107.

4. Bloch PH. Seeking the ideal form: Product design and consumer response. Journal of marketing. 1995;59(3):16-29.

5. Plassmann H, Ramsøy TZ, Milosavljevic M. Branding the brain: A critical review and outlook. Journal of consumer psychology. 2012;22(1):18-36.

6. Wang L, Yang D. How recycling and transformation information influences tourists’ purchase intentions towards green food products. CyTA-Journal of Food. 2025;23(1):2528540.

7. Sun S, Cao J, Wei Y, Yang D, Ke G. Information presentation: enhancing consumer purchase intention through nutritional supplement advertisement design. Frontiers in Nutrition. 2025;12:1602346.

8. Greenhalgh T, Howick J, Maskrey N. Evidence based medicine: a movement in crisis? Bmj. 2014;348.
